# Nearly Complete Genome Sequences of Two Canine *Mamastrovirus 5* Strains from Latin America

**DOI:** 10.1128/mra.01315-22

**Published:** 2023-02-15

**Authors:** Danielle Rodrigues de Deus, Jones Anderson Monteiro Siqueira, Dielle Monteiro Teixeira, Marcelino Antonio Costa Maués, Márcia Janete de Fátima Mesquita de Figueiredo, Edivaldo Costa Sousa, Thayara Morais Portal, Luana da Silva Soares, Hugo Reis Resque, Luciana Damascena da Silva, Yvone Benchimol Gabbay

**Affiliations:** a Section of Virology, Evandro Chagas Institute, Ministry of Health, Ananindeua, Pará, Brazil; b Zoonosis Control Center, Belém, Pará, Brazil; c Federal Rural University of the Amazon, Belém, Pará, Brazil; d Section of Parasitology, Evandro Chagas Institute, Ministry of Health, Ananindeua, Pará, Brazil; e Laboratory of Viral Cell Biology and Therapeutics, Katholieke Universiteit Leuven, Leuven, Belgium; Katholieke Universiteit Leuven

## Abstract

We report the nearly complete genome sequences of CAstV-PK01 and CAstV-PK03, two canine astrovirus strains belonging to the species *Mamastrovirus 5*, which were detected in fecal swab samples collected from puppies with diarrhea from two different kennels in the Brazilian Amazon.

## ANNOUNCEMENT

Canine astroviruses (CAstVs) (*Mamastrovirus 5* species) belong to the *Mamastrovirus* genus, a member of the *Astroviridae* family, which includes nonenveloped, linear, single-stranded, positive-sense RNA viruses (1). In recent years, CAstV strains have been identified worldwide in the feces of animals with symptoms of gastroenteritis (2, 3). Only partial genome sequences of CAstVs from Latin America can be found in GenBank as of 8 August 2022 (4, 5).

Samples (CAstV-PK01 and CAstV-PK03) were obtained in 2019 from a pool of fecal swab specimens from mixed-breed dogs with diarrhea. The animals had been captured in an urban area by public kennels located in the Brazilian Amazon (Belém, Pará, Brazil). The GPS coordinates for sample collection were as follows: CAstV-PK01, −1.315824, −48.454977; CastV-PK03, −1.457762, −48.431807. The fecal swab specimens were washed in 5 mL of 1× phosphate-buffered saline (PBS) and stored at −20°C. Viral RNA was extracted using the PureLink viral RNA/DNA minikit (Invitrogen, Carlsbad, CA, USA) and subjected to cDNA synthesis using the SuperScript VILO synthesis kit (Invitrogen) and the NEBNext mRNA second-strand synthesis module (New England Biolabs, Ipswich, MA, USA). DNA libraries were constructed using the Ion Xpress Plus fragment library kit (Thermo Fisher Scientific, Carlsbad, CA, USA) and sequenced on the Ion Chef system and the Ion GeneStudio S5 system (Thermo Fisher Scientific). The raw reads were obtained with the Ion GeneStudio S5 export plugin and trimmed using the fastp tool v.0.23.1 (6), and *de novo* assembly was performed using MEGAHIT software v.1.2.9 (7) to generate contigs (Table 1). The minimum contig size used as the cutoff value was 200 bp. The contigs were annotated with Kraken2 v.2.1.0 (8), and some were related to members of the *Mamastrovirus* genus (Table 1).

**TABLE 1:** Sequencing and genome metrics

Parameter	Data for strain:	
	CAstV/PK01/2019/BRA	CAstV/PK03/2019/BRA
No. of raw reads	3,611,146	2,588,519
No. of reads after trimming	3,604,976	2,285,222
Avg read length (bp)	151	160
Mean no. of reads after trimming	2,945,099	2,945,099
Total no. of contigs	45,856	162
No. of contigs related to *Mamastrovirus* genus^*a*^	49	1
Genome size (bp)	6,620	6,446
GC content (%)	44.8	45.3
Coverage (×)	3,597.2	860.8
GenBank accession no.	OP093958	OP093957

a These contigs were mapped against a standard database from Kraken2 v.2.1.0, using default parameters (*k*-mer = 35, l = 31).

Visualization and analysis of metagenomic classification results from Kraken2 were performed using the Pavian R package v.1.2.0 (9). The contigs and reads were then mapped against a closely related British reference strain (GenBank accession number NC_026814) and manually curated. All tools were run with default parameters unless otherwise specified.

The open reading frames (ORFs) were predicted using Geneious Prime (10). Both genomes contained a typical genomic organization for astroviruses, with ORF1a, ORF1b, and ORF2. The average read coverage values were 3,597.2× and 860.8× and the GC contents were 44.8% and 45.3% for CAstV-PK01 and CastV-PK03, respectively. The two nearly complete genomes showed 88.39% sequence identity with each other. The CAstV/PK01/2019 and CAstV/PK03/2019 strains showed greatest nucleotide identity (93.8 to 95.1%) with strains from Hungary (GenBank accession number KX599350) and the United Kingdom (GenBank accession number KP404150), respectively.

CAstV/PK01/2019 and CAstV/PK03/2019 showed similarity of only 81.18 to 88.39% and 93.62 to 94.58%, respectively, with respect to the available partial genomes of Brazilian CAstV strains (GenBank accession numbers JN705937, JX878479 to JX878484, KC470086 to KC470089, KR349488 to KR349491, and KY765684). Strains CAstV/PK01/2019 and CAstV/PK03/2019 are thought to belong to the *Mamastrovirus* genus because they are closely related to strain Gillingham/2012/UK (GenBank accession number NC_026814), which belongs to the *Mamastrovirus 5* species.

The use of the metagenomic approach provided evidence for the presence of these viruses in dogs that showed symptoms of diarrhea and were captured on the streets, posing a risk of infection to other animals that live in public areas. Consequently, comprehensive epidemiological and molecular investigations are necessary, specifically for CAstV strains in dogs with or without diarrhea, for effective control of CAstV infection.

### Data availability.

The sequences were deposited in GenBank under the accession numbers OP093957 and OP093958. The accession numbers for the Ion Chef system sequencing raw reads in the NCBI Sequence Read Archive (SRA) are BioProject accession number PRJNA909764, BioSample accession numbers SAMN32093330 and SAMN32093331, and SRA accession numbers SRR22561741 and SRR22561740.
